# Machine learning applied to mild cognitive impairment: bibliometric and visual analysis from 2015 to 2024

**DOI:** 10.3389/fneur.2025.1587441

**Published:** 2025-05-21

**Authors:** Huan Liu, Qing Huo, Feng Li, Xu Luo, Renli Deng

**Affiliations:** ^1^Department of Nursing, Affiliated Hospital of Zunyi Medical University, Zunyi, China; ^2^School of Nursing, Zunyi Medical University, Zunyi, China; ^3^Maternal and Child Health Hospital, Zunyi, China; ^4^School of Medical Information Engineering, Zunyi Medical University, Zunyi, China

**Keywords:** machine learning, mild cognitive impairment, Citespace, VOSviewer, visual analysis

## Abstract

**Background:**

At present, the world is in the background of severe aging population challenges. Mild cognitive impairment (MCI), an intermediate state between normal aging and dementia, is a syndrome of cognitive impairment. Early recognition and intervention of MCI have great value for delaying the decline of cognitive function and improving the quality of life in the elderly. Machine learning (ML) is the core sub-branch direction in the field of artificial intelligence. In recent years, evaluating the potential application of machine learning in medicine has been popular, including the field of mild cognitive impairment. However, there is currently no bibliometrics to evaluate the scientific advances in this field.

**Objective:**

This study aims to visually analyze the current research trends regarding the application of machine learning in the field of MCI through bibliometry and visualization techniques.

**Methods:**

Using the Web of Science Core Collection database (Wo SCC), relevant articles and reviews of the collection database 2015–2024. Subsequently, the collected papers were subjected to bibliometric analysis utilizing CiteSpace, VOSviewer, and the “bibliometric” package in R language.

**Results:**

A total of 2056 papers related to machine learning in patients with MCI were retrieved from the Wo SCC database. The number of papers is increasing year by year. These papers are mainly from 9,577 organizations in 498 countries, most of which are from the United States and China. The journal with the largest number of publications is the FRONTIERS IN AGING NEUROSCIENCE. Folstein M is an authoritative author from the Johns Hopkins University School of Medicine. His paper “Mini-mental state: A practical method for grading the cognitive state of patients for the clinician” is the most cited article in this field. Literature and keyword analysis indicate that MCI prediction, automated monitoring of MCI, continuous evaluation and remote monitoring of cognitive function in individuals with MCI, and interdisciplinary data integration and personalized medicine are current research hotspots and development directions.

**Conclusion:**

This study is the first to use bibliometric methods to visualize and analyze the application field of machine learning in MCI, revealing research trends and frontiers in this field. This information will provide a useful reference for researchers focusing on machine learning applications in the field of MCI.

## 1 Introduction

The early stage of dementia is mild cognitive impairment (1), which can be specifically manifested as one or more function decline in memory, executive function, language, application, and visual spatial structure skills, which can lead to the corresponding clinical symptoms, but the ability of daily living activities is basically normal, and does not meet the clinical diagnosis criteria of dementia. At present, the global prevalence of MCI is about 6.8–32% (2–4). If the patient with mild cognitive impairment further deteriorates to the dementia stage, the patient has late loss of independent living, which will bring heavy economic burden to the society and family (5). Therefore, the focus on this stage has become a key entry point to delay the development of dementia.

Artificial intelligence is a branch of computer science, and machine learning is an important research direction in the field of artificial intelligence research. As a data analysis tool, machine learning has the ability to extract and analyze a large number of different types of clinical data sets. In addition, machine learning is also applied to assist diagnosis, disease triage, disease risk prediction and rapid disease identification (6, 7). In the field of mild cognitive impairment, machine learning technology is good at processing multimodal and high dimensional data, can be regarded as a reliable tool (8–13) for early differential diagnosis and prediction of disease progression of cognitive decline in the elderly, thus, machine learning plays a crucial role in the field.

Notably, an increasing number of articles on MCI and machine learning are published each year. Therefore, it is indispensable for researchers to keep continuous attention to the latest literature in this field and a comprehensive timely update of information through dynamic visualization. Bibliometric analysis is a statistical method based on public literature databases, not only allowing the quantitative and qualitative evaluation of publications to help in the analysis of domain-specific research hotspots and trends (14), And bibliometrics can help decision-making and research manage (15) by providing new information. In recent years, the application of machine learning in cognitive impairment research developed rapidly, in addition to the lack of bibliometric analysis associated with mild cognitive impairment, it is noteworthy that the multidisciplinary nature of the field and its subject diversity prompted us to machine learning in mild cognitive impairment related literature to conduct a comprehensive macro analysis. Therefore, the main purpose of this study is to conduct bibliometric and visual analysis of the application of machine learning in MCI in the past 10 years, to determine the main research status and research prospects of this field, and to provide new ideas and reference significance for future research problems.

## 2 Materials and methods

### 2.1 Data sources and collection

**Figure 1:**
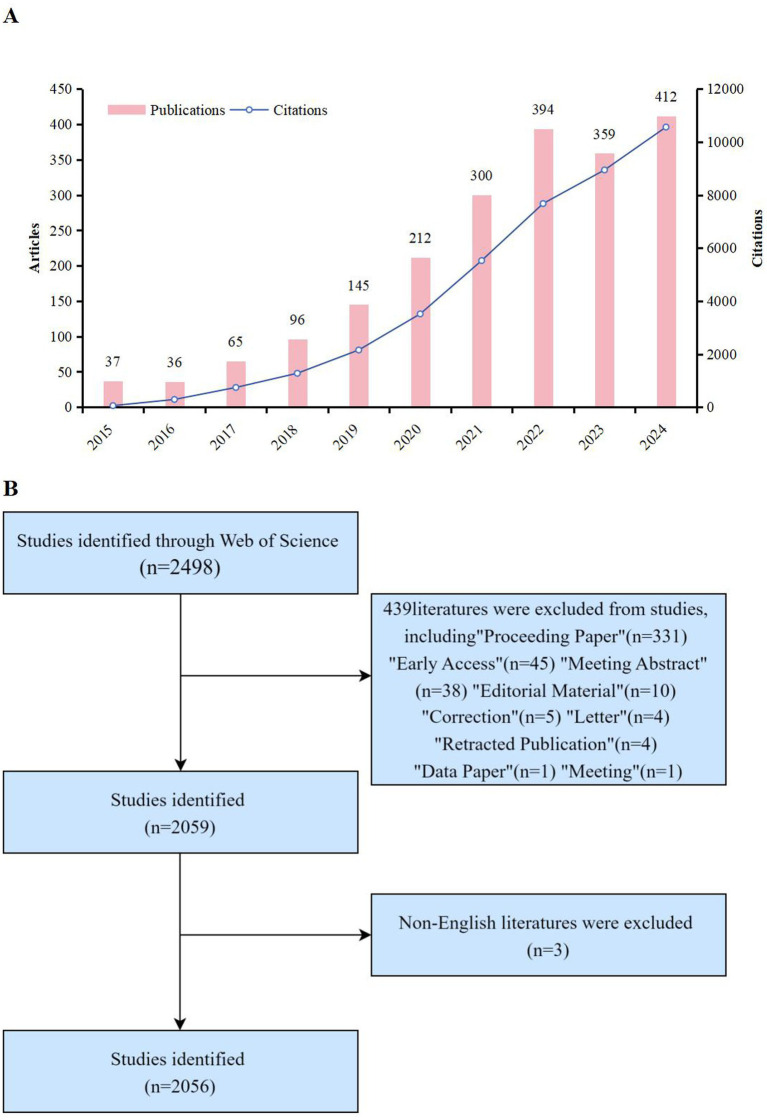
**(A)** Flowchart and annual results of paper screening for ML in Mild Cognitive Impairment research, 2015—2024. **(B)** Annual publication volume and annual citation frequency of relevant articles in the past 10 years (2024 data, as of December 31).

The bibliometric analysis data for this study are from the Web of Science Core Collection (Wo SCC), which is known for its comprehensive and authoritative collection of research publications and is a good bibliometric analysis database for its rich analytical indicators that enable researchers to identify research hotspots and trends in their respective fields. In Wo SCC (16), TS represents the topic statement. The retrieval form used in this study was set to (((((TS = (“Cognitive Dysfunctions”)) OR TS = (“Cognitive Disorder *”)) OR TS = (“Cognitive Impairment *”)) OR TS = (“Mild Cognitive Impairment *”)) OR TS = (“Cognitive Decline *”)) OR TS = (“Mental Deterioration*”)) AND TS = (“Machine Learning”). The search time was limited from 01 January 2015 to December 31, 2024. The selection was limited to “articles” and “review articles” in English, resulting in a total of 2,056 publications (Figure 1A). According to the above formula, the results were exported as plain text files in txt and csv formats for the search on Wo SCC. The search was completed on 01 January 2025 to prevent data bias due to database updates. Then two researchers (QH and FL) verified the assessment separately. Any discrepancies were taken for reassessment by a third party and immediately followed by a three-way harmonization.

### 2.2 Data analysis and visualization

CiteSpace Software system (17) is an information visualization software developed by Dr. Chaomei Chen, a Chinese scholar from the School of Information Science and Technology, Drethel University, mainly for the measurement and analysis of scientific literature data. We used CiteSpace 6.4.Rl (64-bit) Advanced and VOSviewer1 version 6.20 visualization software to analyze national or regional distribution and collaboration, institutional collaboration and publication volume, author distribution and collaboration, biplot superposition of journal and keyword analysis, and co-cited reference collaboration. VOSviewer is a visualization tool specially developed to analyze the knowledge units of the literature by van Eck et al. (18) based on VOS visualization technology. Its outstanding advantage is its strong graphics display ability, which is suitable for analyzing large-scale sample data, and can cluster the knowledge units. Based on the search results in the Web of Science database, the raw data is processed and imported into VOSviewer, whose results are presented in the form of a visual atlas.

### 2.3 Research ethics

All raw data used in this study were obtained from the Web of Science public database, and all data sources used were publicly available, so no ethical review was required.

## 3 Results

### 3.1 Global overview

Through our search and filtering endeavors, we identified a total of 2,056 articles, which included 1,847 articles and 209 reviews. The average article age was 3.49, each author publishes an average of 7.78 articles. Moreover, the average number of citations per article was 19.97, this may be relevant to the newer situation in this field. In general, 12,413 authors from 498 regions and countries have published relevant literature on ML application to mild cognitive impairment in 609 journals worldwide.

### 3.2 Analysis of annual publication and citation trends

Figure 1B illustrates the annual count of publications and the frequency of citations for relevant articles over the past decade (2015–2024). Overall, there is a discernible upward trajectory in the annual publication count concerning the application of machine learning in mild cognitive impairment. We conducted an analysis of the annual article count from 2015 to 2022, observing a steady increase in the number of publications. A more pronounced surge in publications occurred after 2020. The annual citation frequency and the number of published papers reached 5,526 and 300, respectively, by 2021. However, the number of publications has declined during the year 2023. By 2024, the number of published articles and the annual citation frequency had risen to 10,561 and 412, respectively, before continuing on its significant increasing trajectory. We can conclude that more scholars might become interested in this topic in the future based on these general publishing trends.

### 3.3 Distributions of countries/regions

Presently, 498 countries/regions have participated in the application of ML in mild cognitive impairment. The top 10 countries have a large proportion of publication output in this area (Table 1). The United States has the largest number of publications (2,748 articles), citations (1,0,011 articles), and joint publications (multinational publications: 129) in the discipline. The United States and China are the main forces in applied application of ML in mild cognitive impairment. China and the United Kingdom are ranked second and third in the number of publications. Although the number of publications in the UK and France lags with the US and China, their international cooperation ratio (French MCP ratio: 64.3%; UK MCP ratio: 62.6%) is high. In general, most countries have exchanges and cooperation, while countries such as Canada and India currently have a low proportion of international cooperation. The lack of international cooperation in the field of mild cognitive impairment and machine learning may limit the research and development of mild cognitive impairment and machine learning, which should be addressed in the future.

**Table 1:** The top 10 productive countries.

Rank	Country	Articles	Citations	SCP	MCP	MCP %
1	USA	2,748	10,011	325	129	28.4
2	China	2,205	5,931	347	115	24.9
3	United Kingdom	558	4,618	40	67	62.6
4	Italy	519	2,109	60	35	36.8
5	Canada	507	1882	63	22	25.9
6	South Korea	500	1807	69	37	34.9
7	Germany	468	1,528	27	27	50
8	Spain	369	1,681	49	29	37.2
9	India	244	1,203	76	20	20.8
10	France	226	1,201	15	27	64.3

SCP, single country publications; MCP, multiple country publications.

**Figure 2:**
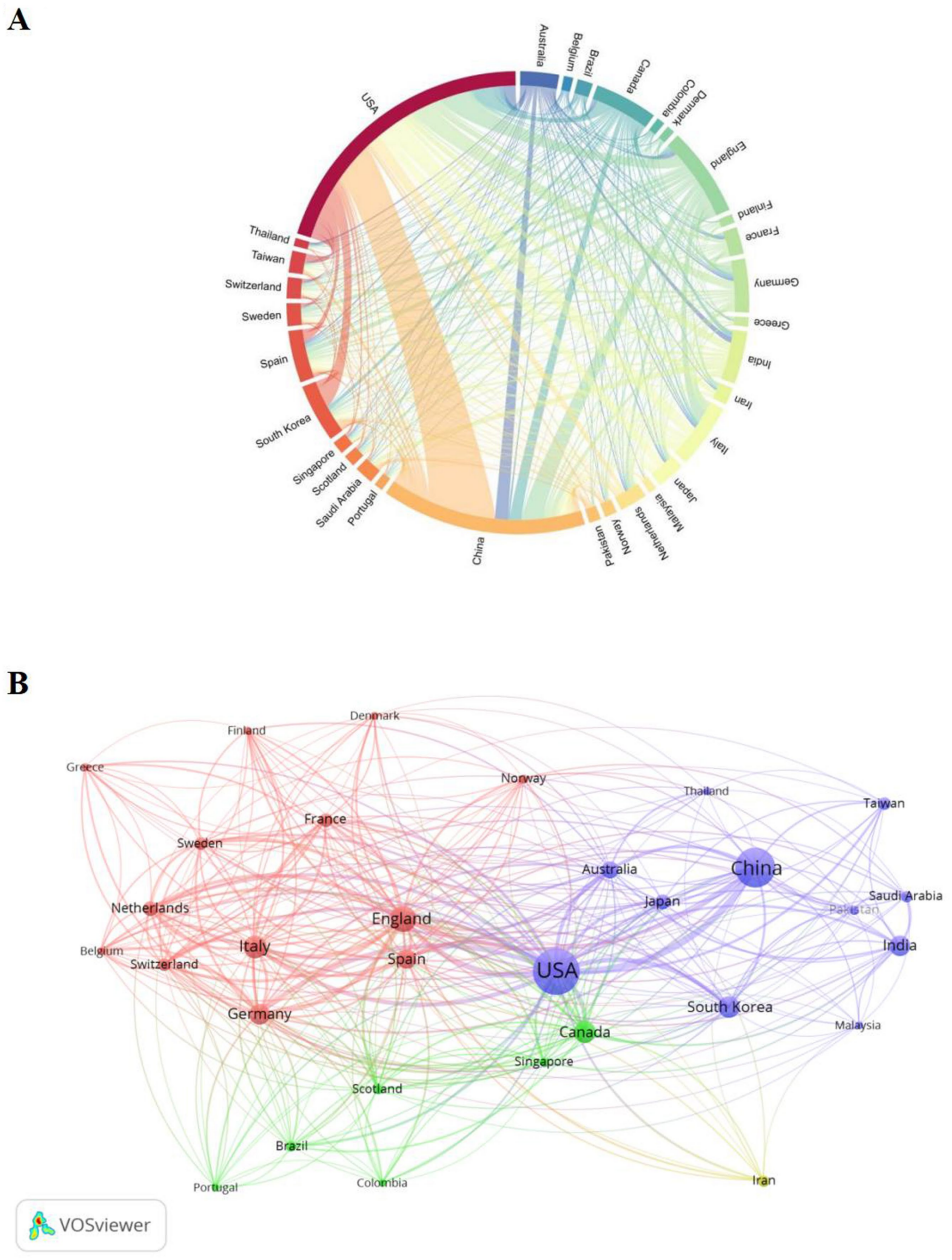
Machine learning and MCI related countries regions analysis. **(A)** Chord plot of country region cooperation. Each external curve represents a country region, and the thickness of the line is directly related to the strength of cooperation between countries/regions. **(B)** Visualization of the cooperation network between countries regions using VOSviewer. The figure shows the top 30 countries/regions in terms of the number of documents. Nodes of different colors represent different clusters of countries regions, and the size of the nodes corresponds to their respective saliency.

Furthermore, we delineated the top 30 countries/regions in terms of the number of publications in this area on a country cooperation map (Figures 2A,B). A chord diagram (19, 20) is a graph that can be used to show relationships and flows between different entities. Each sector in (Figure 2A) represents a country, and the thickness of the string is usually proportional to the strength of the cooperation. The wider sectors in China and the United States may indicate that both countries have published or influential publications in the field of machine learning assistance in the treatment of MCI. As can be seen from the figure, the United States has extensive cooperative relations with many countries, especially with China, Canada and Germany. This suggests that the United States is a central hub in international research cooperation. In addition, China also works with many countries, especially Australia, South Korea and Japan, which shows that China is an important partner in the application of machine learning in the field of MCI research. European countries, such as the United Kingdom, Germany, France, Italy, etc. have formed a relatively intensive cooperation network in the map, showing the activity of scientific research cooperation between European countries and with other countries. For example, Japan, South Korea, Singapore and other Asian countries have small sector areas, but they also show certain cooperation networks in the figure, and their contributions in the field of cognitive impairment cannot be ignored. By the VOSViewer, Figure 2B shows the collaboration between countries involved in research on the application of ML in mild cognitive impairment with the top 30 publications. Each node represents a country, the size of the node is usually proportional to the activity of the country in the cooperative network, and the connection between the nodes indicates the academic cooperation between the two countries (21). Different colors represent different clusters. The purple groups include the United States, China, South Korea, Japan and Australia, while the red groups include Italy, France, Germany, Spain, Sweden and other countries; The green group covers Brazil, Portugal, Colombia, Scotland and other countries. In the figure, Iranian nodes are yellow and less connected with other countries, which may indicate a more independent partnership in Iran in the cooperative network.

### 3.4 Distributions of institutions

Presently, a total of 9,577 institutions are involved in research on the application of ML in mild cognitive impairment. Table 2 shows the top 10 institutions in terms of number of publications. The institution with the highest number of publications is Harvard Medical School (53), followed by the Chinese Academy of Sciences (43). Among the top 10 institutions in terms of the number of publications, four are from the United Kingdom, followed by three from the United States and two from China, one is originally from Canada. Significantly, Total link strength is a measure of the strength of partnerships between institutions. The higher the link strength, the closer the cooperation between the two institutions. Harvard Medical School (279) is higher than the Chinese Academy of Sciences (222), which means that these institutions have an important position to study the application of machine learning in the field of mild cognitive impairment.

**Table 2:** The top 10 productive institutions.

Rank	Institution	Publications	Citations	Total link strength
1	Harvard Medical School	53	1,107	279
2	Chinese Academy of Sciences	43	1,239	222
3	University of Pennsylvania	40	1831	205
4	University College London	35	1,287	181
5	Capital Medical University	33	478	157
6	University of Toronto	32	1,242	142
7	Stanford University	28	583	153
8	University of Cambridge	28	1,189	151
9	Imperial College London	27	1846	147
10	King’s College London	27	1720	177

**Figure 3:**
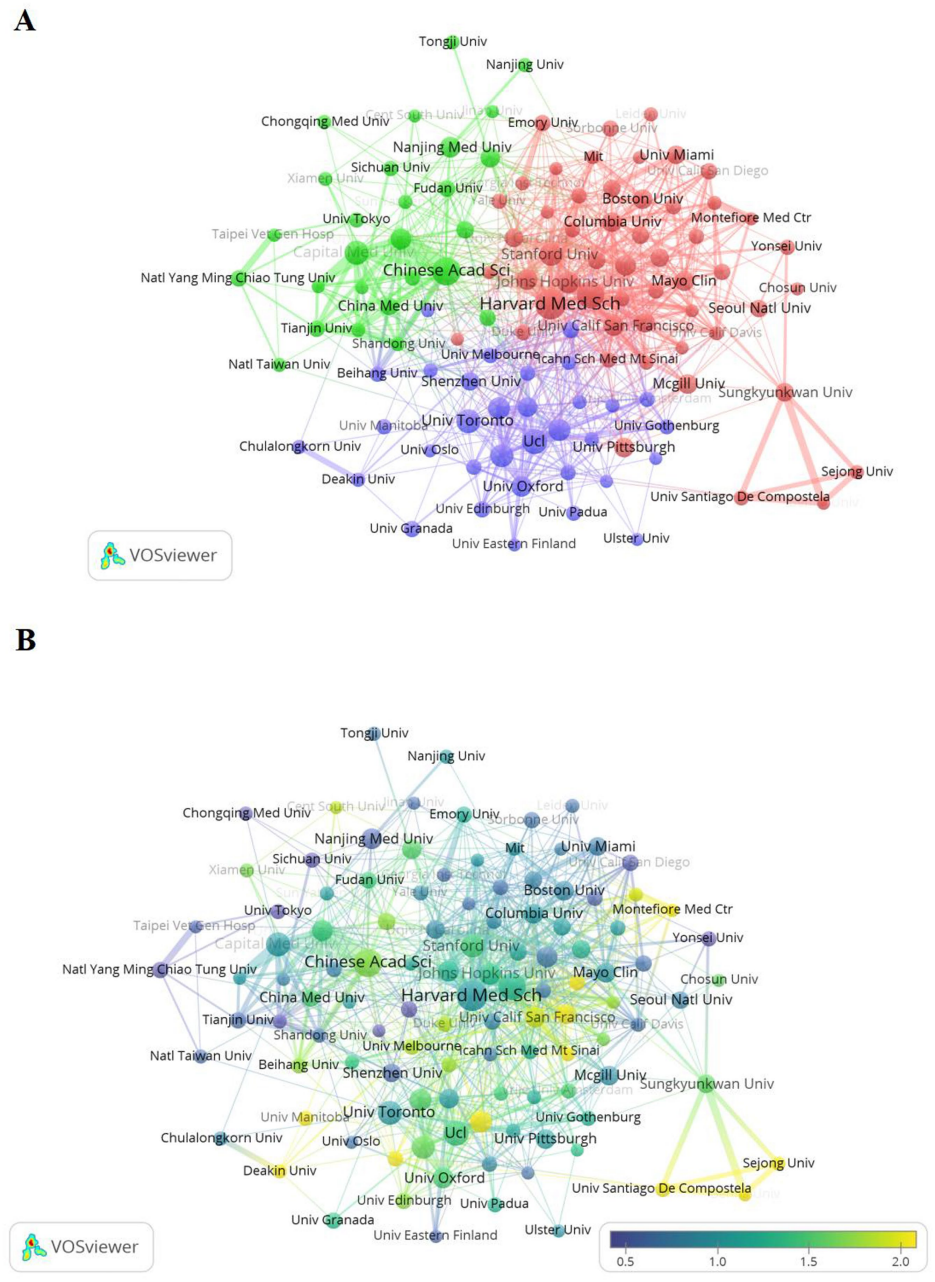
Analysis of network-related institutions. **(A)** Visual analysis of the institutional collaboration network in VOSviewer. Institutions with more than 10 publications are shown in Fig. Nodes of different colors represent Institutions in different color clusters, and the size of the nodes indicates the frequency of their occurrence. **(B)** Visual analysis of institutional average normalized citations in VOSviewer.

The purpose of the analysis for research institution (22) was to understand the global distribution of research related to ML in mild cognitive impairment and to provide new opportunities and probability for collaboration. In VOSviewer, we demonstrated the top 113 institutions with more than 10 publications and categorized collaborations between institutions into three highly correlated clusters (Figure 3A).

Figure 3B shows the average citations for institutional publications. The closer the color is to yellow, according to the legend, may indicate that these institutions have a high influence in the research field of machine learning and MCI. Conversely, if the color is biased toward purple, it indicates a lower proportion of average citation, indicating that these institutions have had relatively little publications or influence in this area in recent years. According to the exhibition, Harvard Medical School is located in the center with large nodes, indicating its core influence in the cooperation network. The Chinese Academy of Sciences is also located in the center with large nodes and warm colors, so its influence cannot be ignored. Institutions such as Imperial College London and University College London have smaller nodes than those of Harvard Medical School, but it is also worth learning from their advanced research techniques.

### 3.5 Distribution of authors

Table 3 shows the top 10 authors in terms of number of publications and co-citation frequency. Authors collaborative network analysis (23, 24) is indispensable in academic research because it can help identify key researchers, research teams and collaborative groups, understand research dynamics within the discipline, and discover potential collaborative opportunities. Table 3 shows that there are five from the United States, two from China, among them, the top three authors publications as much (all 13 articles), respectively is in the United States in Framingham heart research Rhoda Au, working at the university of Pennsylvania Pererelman school of medicine Christos Davatzikos and Ying, Han, Shanghai, China. Co-citation author analysis (25, 26) is the phenomenon that two or more authors are cited simultaneously cited in the same literature, which may indicate that these authors share similar research topics or fields. As can be seen in Table 3, the authors with the highest cocited frequency in this field are Jack, C. R from the Department of Neurology in Rochester, Minnesota, and Petersen, R. C from the Alzheimer’s Disease Research Center at Mayo Clinic.

**Table 3:** Top 10 authors in number of publications and co-citations.

Rank	Author	Publications	Total link strength	Author	Co-citations
1	Rhoda Au	13	89	Jack, C.R	694
2	Christos Davatzikos	13	120	Petersen, R.C	558
3	Ying, Han	13	164	Folstein, M.F	262
4	Beheshti, Iman	12	41	Fischl, B	261
5	Shaker, El-Sappagh	10	45	Breiman, L	246
6	Mohamad Habes	10	96	Suk, H.I	222
7	Dinggang,Shen	10	60	Dubois, B	220
8	Frederik Barkhof	9	73	Zhang, D.Q	216
9	James H,Cole	9	70	Morris, J.C	209
10	Galvin, James E	9	50	Cole, J.H	195

**Figure 4:**
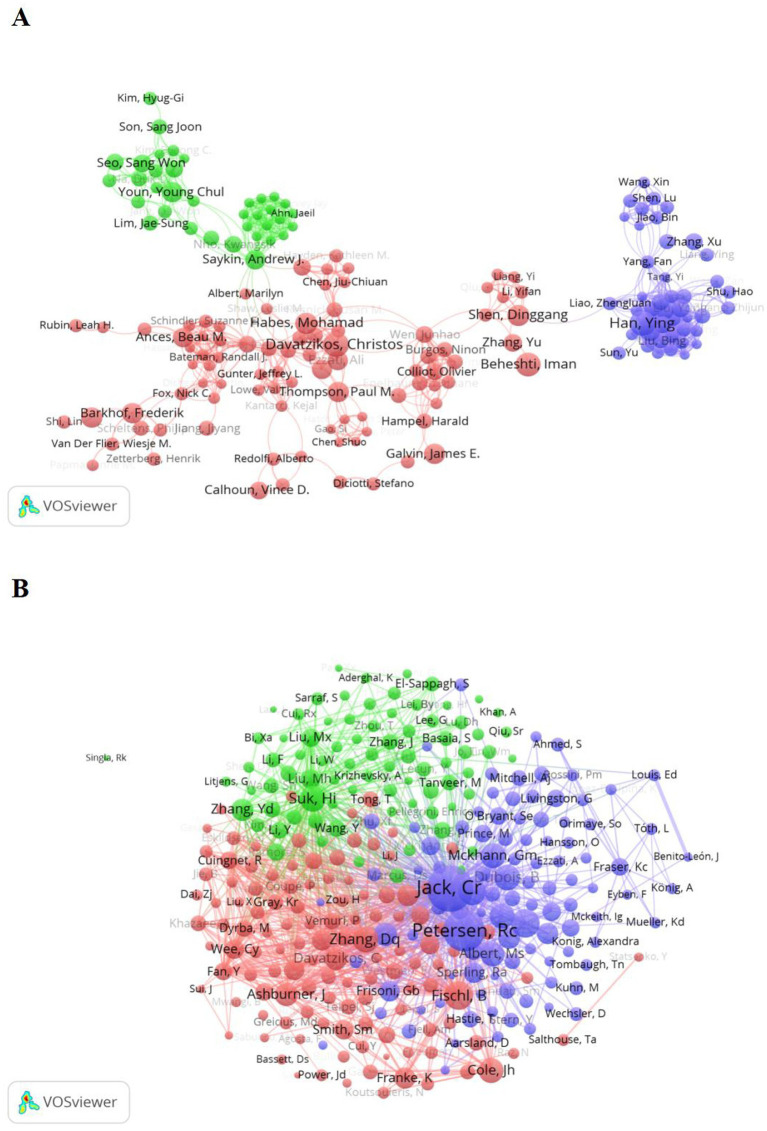
Author analysis in relation to machine learning and MCI studies. **(A)** Collaborative network analysis of the top 300 authors in the publication. Nodes of different colors represent authors in different color clusters, and the size of the nodes usually represents the number of published articles or the frequency of collaboration between authors.**(B)** The authors of machine learning in MCI-related studies co-cited the network analysis. The size of the node reflects the number of citations or effects of the author.

With the help of VOSViewer on the field of machine learning and mild cognitive impairment of the top 300 authors collaborative network analysis, through Figure 4A, the top 300 authors according to the closeness of collaboration is divided into three main blocks, the size of the node usually represents the number of published articles or the frequency of collaboration between the authors, the results are presented in different colors. The red block mainly includes the Davatzikos, Christos, Habes, Mohamad, Ances, Beau-M, et al. The purple block mainly includes the HanYing, LiuYong, YangFan, et al.; while the green block mainly includes the Saykin, Andrew J, Nho, Kwangsik, et al. It is worth noting that in the green block, Saykin, Andrew J have been associated with multiple other authors, which may mean that he plays an important role in the field under study or has an extensive cooperative relationship.

Figure 4B shows the network map of co-cited author relationships. According to the figure, the red cluster mainly includes Fischl, B, Cole, Jh, Zhang, Dq, Davatzikos, Christos, etc., the authors of the largest nodes in the green cluster are Suk, Hi, and other authors mainly include Zhang, Yd, Liu, Mx, etc. The authors of the two largest nodes of the purple cluster are Clifford R. Jack Jr. and Petersen Rc. The high citation number of these two authors indicates that their research results have extensive influence in academia and have a profound impact on the field of cognitive function (consistent with the popularity of authority in actual research).

### 3.6 Distribution of journals

In our careful analysis, 609 journals have published papers on mild cognitive impairment and machine learning. Table 4 shows the top 10 journals by the number of publications, and the top 10 journals are all journals with more than 30 published articles. H-index (27, 28) helps to assess the consistency and influence of journal publication of highly cited articles. As shown in the figure, the most frequent publications were “FRONTIERS IN AGING NEUROSCIENCE” (102) and “JOURNAL OF ALZHEIMERS DISEASE” (97), and both journals topped the H-index: “FRONTIERS IN AGING NEUROSCIENCE” (H-index: 20), “JOURNAL OF ALZHEIMERS DISEASE” (H-index: 20), Shows its importance and contribution to the field. The number of publications followed by H-index are “SCIENTIFIC REPORTS” (TP: 62; H-index: 19) and “FRONTIERS IN NEUROLOGY” (TP: 45; H-index: 19). In addition, the journal with the highest total citation volume was “SCIENTIFIC REPORTS” (3197).

**Table 4:** Top 10 journals by number of publications.

Rank	Journal	Country	TP	TC	H-index	IF (2023)
1	FRONTIERS IN AGING NEUROSCIENCE	Switzerland	102	1793	20	4.1/Q2
2	JOURNAL OF ALZHEIMERS DISEASE	Netherlands	97	1794	20	3.4/Q3
3	SCIENTIFIC REPORTS	UK	62	3,197	19	3.8/Q2
4	FRONTIERS IN NEUROLOGY	Switzerland	45	1,047	19	2.7/Q3
5	FRONTIERS IN NEUROSCIENCE	Switzerland	40	1,136	18	3.2/Q3
6	ALZHEIMERS RESEARCH & THERAPY	UK	36	676	16	8.0/Q1
7	IEEE ACCESS	USA	35	1,046	15	3.4/Q3
8	NEUROIMAGE	Netherlands	31	678	14	4.7/Q2
9	HUMAN BRAIN MAPPING	USA	30	968	14	3.5/Q2
10	PLOS ONE	USA	30	684	14	2.9/Q3

TP, total publications; TC, total citations.

**Figure 5:**
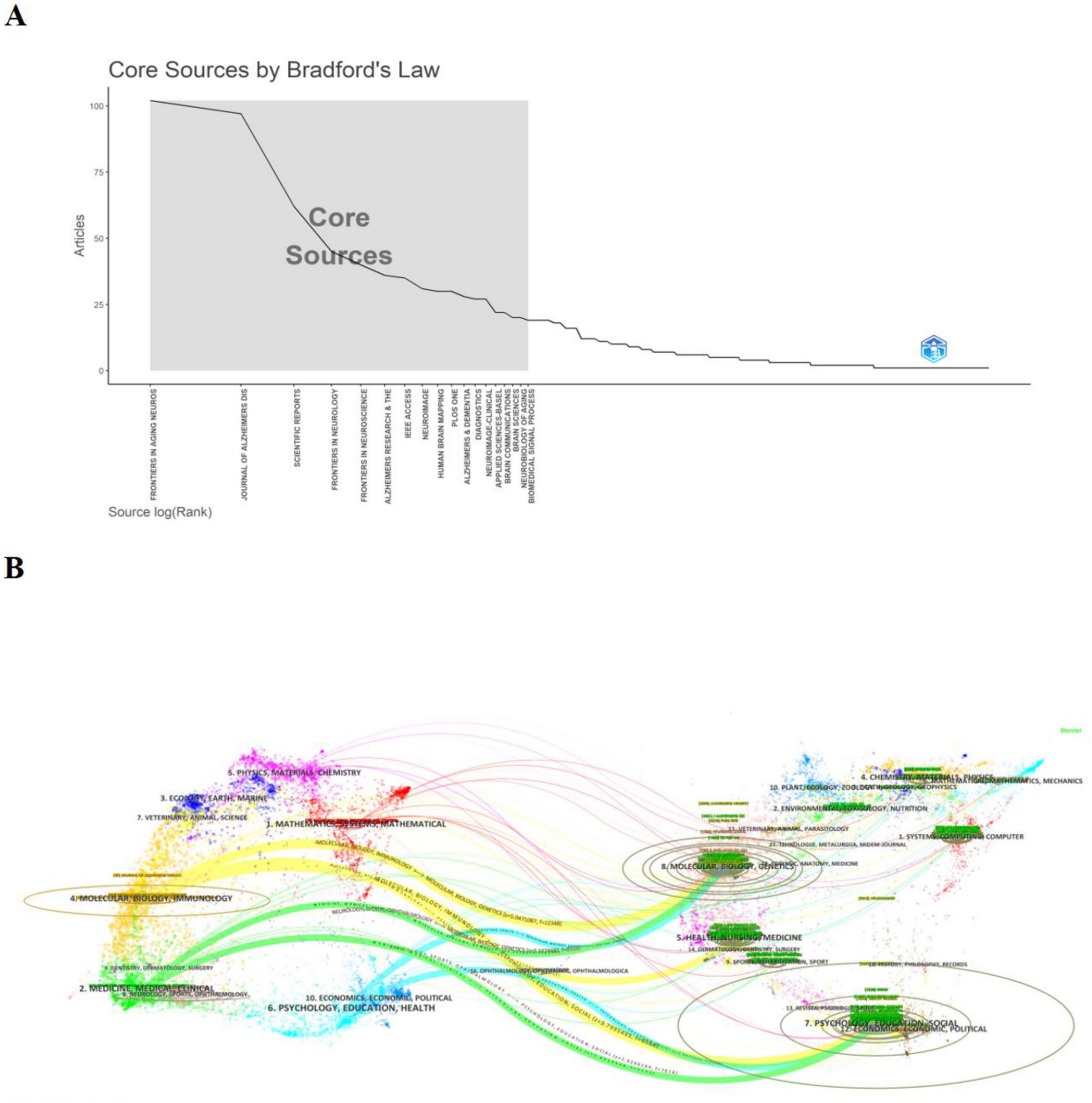
Journals analysis in relation to machine learning and MCI studies. **(A)** Bradford’s law graph for journals related to the application of machine learning m MCI. **(B)** Dual-graph overlay of journals. Citing journals are shown on the left, cited journals on the right, and colored paths represent citation relationships.

Bradford’s law (29) can show the distribution of articles in different journals within a particular field, helping to identify the core journals in the field. Figure 5A shows the number of articles published in the core journals in the field as 508, representing 24.7% of the total articles. Ultimately, the chart of Bradford’s Law identifies 18 core journals on machine learning applications in the field of MCI (2015–2024).

To reveal interdisciplinary crossover and track progress in the frontiers of science, we performed a biplot superposition analysis using CiteSpace to visualize the relationship of citations between journals (30). As shown in Figure 5B, the citing journals are on the left, and the cited journals are on the right. We can see the most important seven paths. The topics of Citing Journals are mainly SYSTEMS, MATHEMATICAL, MEDICINE, MEDICAL, CLINICAL namely research frontier. The topics of Cited Journals are mainly SYSTEMS, COMPUTER, ENVIRONMENTAL, TOXICOLOGY, NUTRITION, CHEMISTRY, MATERLALS, PHYSICS known as the knowledge base.

### 3.7 Keyword analysis

Table 5 shows the top 10 keywords in order of frequency of occurrence. The most frequent keyword was “machine learning” (1,017), followed by “Mild Cognitive Impairment” (855). Furthermore, “Dementia” (697), “Alzheimer’s Disease” (617), “Classification” (369), “Diagnosis” (330) are also the keywords with high frequency, indicating that these keywords are popular with researchers in their research field.

**Table 5:** Top 10 keywords in the field of MCI and machine learning.

Rank	Keyword	Occurrences	Total link strength
1	Machine Learning	1,017	8,736
2	Mild Cognitive Impairment	855	7,732
3	Dementia	697	6,323
4	Alzheimer’s Disease	617	5,525
5	Alzheimers-Disease	473	4,253
6	Classification	369	3,439
7	Diagnosis	330	3,009
8	Cognitive Impairment	298	2,398
9	Mri	241	2,313
10	Prediction	207	2022

**Figure 6:**
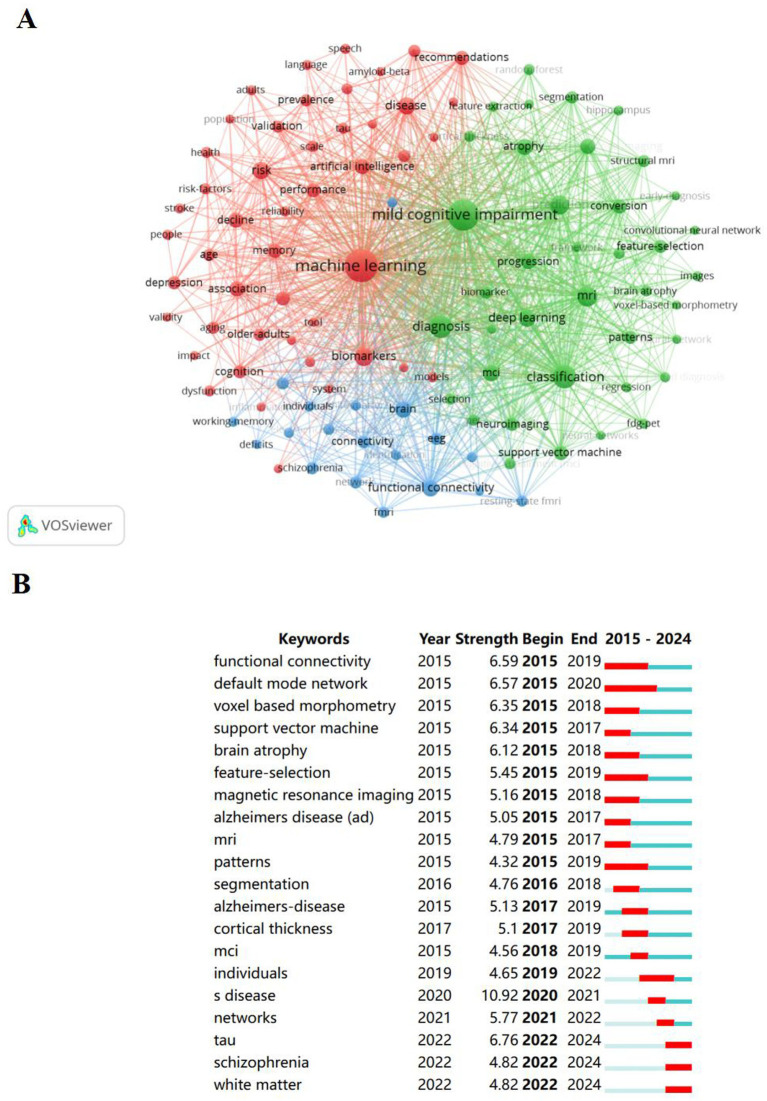
Keywords analysis in relation to machine learning and MCI studies. **(A)** Collaborative network visualization of keywords in VOSviewer. Nodes of different colors indicate keywords of clusters of different colors, and the size of nodes indicates their frequency. **(B)** Top 20 Keywords with the Strongest Citation Bursts.

Figure 6A is the keyword co-occurrence network of machine learning in the MCI research field, showing the keywords appearing more than 20 times. Three clusters were obtained through VOSviewer. Red 1 cluster for “MCI prediction by biomarkers, artificial intelligence and machine learning”; green 2 cluster for “early diagnosis of MCI”; blue 3 cluster for “early scientific intervention and MCI progress monitoring in the field of MCI research.” First, in addition to showing the current situation of MCI epidemiology, red cluster also shows that machine learning plays an extremely important role in the field of mild cognitive impairment and is a popular research topic. The keywords of red number 1 cluster mainly include “prevalence,” “artificial intelligence,” “risk-factors,” “recognition,”“model,” “tool,” “validity,” “machine learning” (31–34). Notably, the 2 cluster theme demonstrates the importance of early accurate diagnosis of patients with MCI and the promotion of cognitive health in the elderly. The terms in the Green Number 2 cluster are mainly covered “early-diagnosis,” “computer-aided diagnosis,” “fdg-pet,” “magnetic resonance imaging,” “structural mri,” “neuroimaging,” “pet,” “mild cognitive impairment” (35–37). Cluster 2 shows that the development of more accurate cognitive assessment tools may also be a mainstream direction in the field of MCI. Finally, the blue 3 cluster shows research directions for exploring scientifically feasible ways and advanced technologies to better monitor the development of the outcome of MCI disease, with the main terms are “electroencephalography,” “individuals,” “identification,” “ognition.”

To further analyze the hotspot temporal dynamics of keyword significance, we extracted the citation frequency of all keywords using the CiteSpace software, and finally obtained the top 20 burst keywords from the data. The most cited keywords are “machine learning” and “mild cognitive impairment.” It is worth noting that since 2022, the popularity of keywords “tau,” “schizophrenia” and “white matter” has continued to rise. In addition to practical research and literature reports, these three keywords may also be related to the occurrence and development of MCI.

### 3.8 Highly cited reference analysis

According to Table 4, the paper “Mini-mental state”: A practical method for grading the cognitive state of patients for the clinician (38) that published by Folstein MF et al., 1975 in the Journal of Psychiatric Research was the most co-cited paper, with 243 citations. The top 10 most frequently co-cited references are all those with citations above 100.

**Figure 7:**
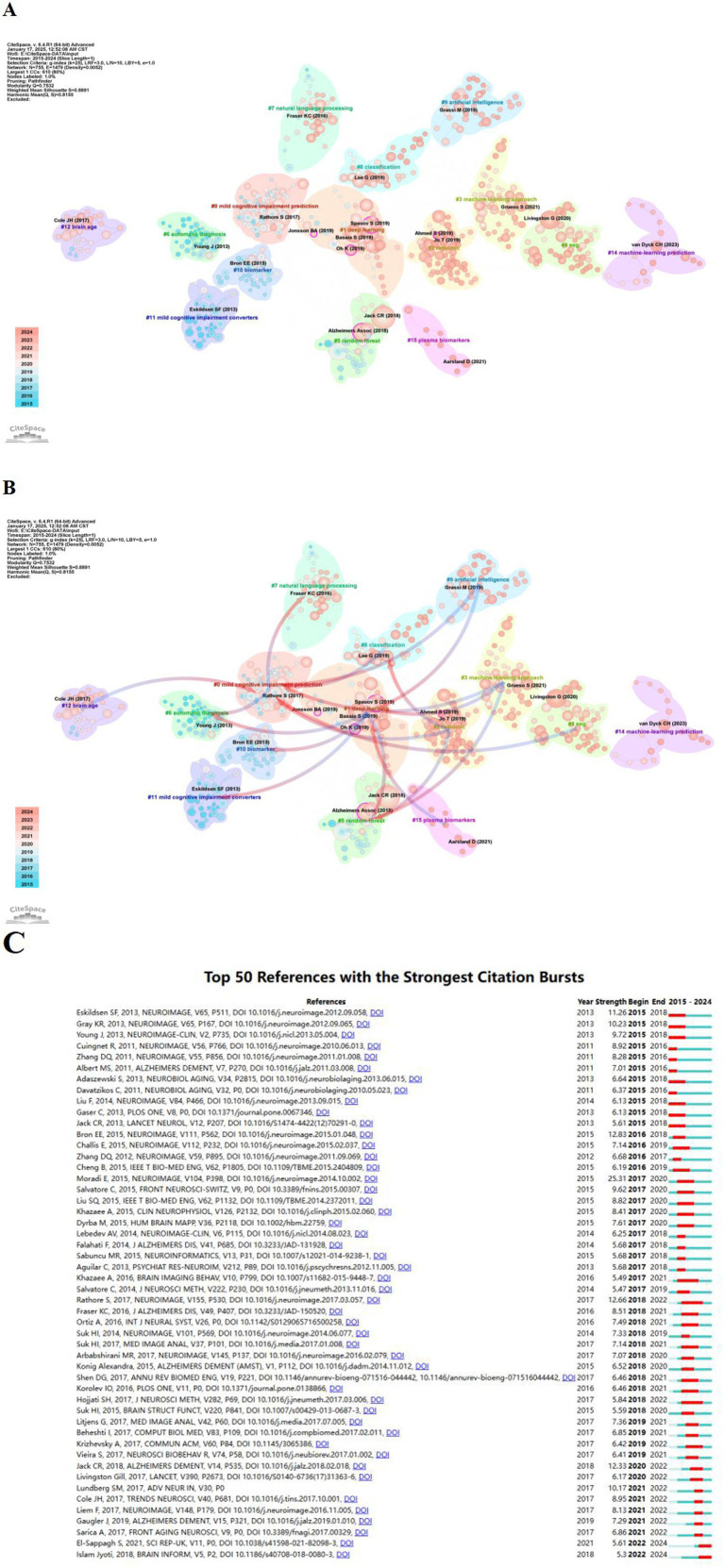
References analysis in relation to machine learning and MCI studies. **(A)** Cluster map analysis of references via CiteSpace. Different color blocks represent different reference clusters. **(B)** The cluster dependency analysis. The blue group is evolved from the red group. References analysis in relation to machine learning and MCI studies. **(C)** Top 50 References with the strongest citation bursts.

Further, the reference cluster analysis and cluster dependency analysis were performed using the CiteSpace 6.4.R1 advanced visualization software, as illustrated in Figures 7A,B. The time span was set from 2015 to 2024, with a time slice of 1 year, focusing on reference nodes. The resulting network comprises 755 nodes and 1,479 links. In Figure 6, the significant cluster structure is denoted by a modularity value (Q value) (39) of 0.7532, and the high confidence level in the clusters by an average profile value (S value) (40) of 0.8891.And the top 10 largest clusters include: “mild cognitive impairment prediction”(cluster #0), “deep learning”(cluster #1), “radiomic”(cluster #2), “machine learning approach”(cluster #3), “eeg”(cluster #4), “random forest”(cluster #5), “automatic diagnosis”(cluster #6), “natural language processing”(cluster #7), “classification”(cluster #8), “artificial intelligence”(cluster #9), “biomarker”(cluster #10). It is very worthy of our attention that the top ranked item by centrality is Oh K (41) in Cluster #1(centrality 0.13). The SpasovS (42) in cluster 1 (centrality 0.12) and the Alzheimer’s Assoc (43) in 5 (centrality 0.12) are the same and rank second together.

On the basis of completing the clustering analysis as described above (Figure 7A), we performed the cluster dependency analysis (44) in Figure 7B. Six red arrows pointing to the “mild cognitive impairment prediction”(#0) has evolved into“radiomic”(#2), “machine learning approach”(#3), “eeg”(#4), “classification”(#8), “artificial intelligence”(#9) and “brain age”(#12). However, the three clusters of “radiomic” (#2), “machine learning methods” (#3) and “artificial intelligence” (#9) have evolved entirely from other clusters, probably suggesting that these clusters are new areas of research. Meanwhile, the burst analysis displayed the top 50 references (Figure 7C). The two latest bursts and continuously emerging references highlight the importance of diagnosis and progression prediction in studies of cognitive impairment and dementia. Together, these studies (45, 46) highlight efforts to reach different stages of identifying dementia through machine learning models or the effects based on interpretable artificial intelligence, and expect superior performance in early diagnosis, thus improving the important clinical understanding of the diagnosis and progression process of cognitive function.

## 4 Discussion

### 4.1 General information

In our study, we use CiteSpace and VOSviewer and Bibliometrix R software to integrate bibliometry and visual analysis, and deeply explore the systematic information of machine learning in MCI research by combining countries/regions, institutions, authors and keywords. Further, stratified analyses were performed based on different countries, institutions, and researchers. On the one hand, readers can help them easily access the knowledge structure and research progress in the field. On the other hand, provide potential partnerships as well as reliable information for researchers and funding agencies.

Using the WoSCC database, we searched for articles published about machine learning and MCI in the present study. The bibliometric study comprised in 2,056 papers from 9,577 institutions in 498 countries/regions with 1,2,413 authors, published in 609 journals. The number of articles over the past 10 years (Figure 1B; January 1,2015 to December 31,2024) has continued to increase overall, with an average citations of 19.97 per article, demonstrating the impact of the field and increasing interest in the use of machine learning in MCI. Although the number of articles has a small inflection point (2022–2023), which may be related to the internal policy adjustment, the number of articles in 2024 returns to an upward trend, exceeds the past number and can be continuously monitored later. The distribution of each country/region (Figures 2A,B) shows that China and the United States have major research relations in the global international cooperation and exchange, and most countries have certain exchanges, however, some countries are relatively low, and the development of MCI into dementia will cause huge medical and social burden. In the future, countries should continue to strengthen international academic exchanges, maintain exchanges in this field and work together to promote their positive development.

As shown in Tables 1, 2 and Figure 2, among the 498 published countries/regions on the application of machine learning in MCI, the United States has the largest number of publications, cited frequency, showing its academic status in this research area. In addition, China and United Kingdom, which ranked second and third in terms of number of publications and frequency of citation, also showed rapid growth. In terms of the number of publications, four of the top 10 institutions were from the United Kingdom, three from the United States, two from China and one from Canada. Harvard Medical School is the highest of all institutions, indicating that it dominates global cooperation in this area. In addition, countries such as China, Canada, Italy and Germany are also widely involved in the research and collaboration of machine learning and MCI.

As can be seen from Tables 3, 6 and Figure 4B, Rhoda Au from the United States is the top author in number of publications, while co-citation frequency is top and far ahead of other authors are Jack, C. R and Petersen, R. C from Rochester, MN, United States, indicating their outstanding impact in application-related areas of machine learning in MCI. It is worth mentioning that the article “Mini-mental state: A practical method for grading the cognitive state of patients for the clinician” (38) by Folstein MF et al. in Psychiatric Research in 1975 is the most frequently cited article in the field. Based on the above data, the analysis shows that Jack, C. R, Petersen, R. C, Rhoda Au and Folstein MF, and their work has had a profound impact on the research in this field. To observe the evolution and development of MCI assessment methods or tools, we also included literature on early cognitive function screening tools related to the MCI theme. Ultimately, our research found that there is indeed a trend: in the early stages, these studies primarily focused on cognitive function screening tools for mild cognitive impairment, such as the Montreal Cognitive Assessment (MoCA) and the Mini-Mental State Examination (MMSE). These assessment tools had not yet incorporated machine learning algorithms, although the authors’ influence was significant. However, over time, many researchers joined the MCI field, and with technological updates and iterations, the impact of MCI research has grown, and the research process has deepened. Eventually, the initial trend of using tools like “MoCA” and “MMSE” to assess individual cognitive function gradually evolved into a subfield of research where machine learning is widely applied to mild cognitive impairment (as shown in the subsequent figures).

**Table 6:** Top 10 highly cited references.

Rank	Author	Article title	Source title	Citations	Year	Document type	DOI
1	Folstein MF et al.	“Mini-mental state”:A practical method for grading the cognitive state of patients for the clinician	PSYCHIATRIC RESEARCH	243	1975	Article	10.1016/0022-3956(75)90026-6
2	Mckhann GM et al.	The diagnosis of dementia due to Alzheimer’s disease: Recommendations from the National Institute on Aging-Alzheimer’s Association workgroups on diagnostic guidelines for Alzheimer’s disease	ALZHEIMERS DEMENTIA	193	2011	Article	10.1016/j.jalz.2011.03.005
3	Albert MS et al.	The diagnosis of mild cognitive impairment due to Alzheimer’s disease: Recommendations from the National Institute on Aging-Alzheimer’s Association workgroups on diagnostic guidelines for Alzheimer’s disease	ALZHEIMERS DEMENTIA	183	2011	Article	10.1016/j.jalz.2011.03.008
4	Jack CR et al.	NIA-AA Research Framework: Toward a biological definition of Alzheimer’s disease	ALZHEIMERS DEMENTIA	156	2018	Review	10.1016/j.jalz.2018.02.018
5	Pedregosa F et al.	Scikit-learn: Machine Learning in Python	MACHINE LEARNING	155	2011	Article	Volume12Page2825–2,830
6	Nasreddine ZS et al.	The montreal cognitive assessment, MoCA:A brief screening tool for mild cognitive impairment	AMERICAN GERIATRICS SOCIETY	146	2005	Article	10.1111/j.1532-5415.2005.53221.x
7	Moradi E et al.	Machine learning framework for early MRI-based Alzheimer’s conversion prediction in MCI subjects	NEUROIMAGE	117	2015	Article	10.1016/j.neuroimage.2014.10.002
8	Zhang DQ et al.	Multimodal classification of Alzheimer’s disease and mild cognitive impairment	NEUROIMAGE	116	2011	Article	10.1016/j.neuroimage.2011.01.008
9	Mckhann G et al.	Clinical diagnosis of Alzheimer’s disease: report of the NINCDS-ADRDA Work Group under the auspices of Department of Health and Human Services Task Force on Alzheimer’s Disease	NEUROLOGY	115	1984	Article	10.1212/wnl.34.7.939
10	Breiman L et al.	Random forests	MACHINE LEARNING	112	2001	Article	10.1023/a:1010933404324

The analysis in Figure 5B shows that in the past 10 years, the main disciplines of interdisciplinary research machine learning at the forefront of MCI generally range from “Physical Sciences/Molecular/Biological/Immunological/Mathematical/Systems” to “Molecular/Biological/Political/Economics/Computer Science/Medical Sciences.” This change means that the research topics in this field are not only focused on the neuropathological mechanisms of MCI, but also to focus on multidisciplinary, multi-field, and society-wide participation in the multidimensional prediction, prevention, and health management of MCI. This interdisciplinary flow and shift helps to drive innovation and address complex problems.

By (Figures 7A, B) show the research theme and direction of machine learning in the emerging field of MCI. Machine learning performs well in the field of mild cognitive impairment and also shows many innovative advantages. Prediction model of mild cognitive impairment: Using different classical machine learning methods, learning to predict MCI when different types of neurocognitive metadata (47) are used as inputs. Machine learning can detect changes in brain structure and train the model to identify early signs of change. The emergence of novel cross-converter models (48) predicts MCI from speech, language, and visual data, allowing multimodal integration to improve the early detection of mild cognitive impairment. Research in the field using convolutional neural networks is also increasing, and some studies have developed a novel deep learning method (49) using functional near-infrared spectroscopy (50) for prediction. Machine learning used to quickly and accurately identify individual mild cognitive impairment is crucial.

Automated monitoring of mild cognitive impairment: traditionally, diagnostic methods such as neurocognitive tests, blood tests, and spinal fluid analysis are laborious, time-consuming, and error-prone. At present, the number of automatic EEG signal analysis using machine learning technology and automatic MCI diagnosis based on EEG (51) has increased repeatedly. Using machine learning techniques to use natural language processing (52) to reveal individual cognitive states from recordings and automatically detect mild cognitive impairment is a relatively applicable approach to real-world environments. In addition to speech-based, machine learning to develop facial expression recognition algorithms (53) in facial videos (54), improving the accuracy of MCI detection.

Continuous evaluation and remote monitoring of cognitive functions of MCI individuals: Some wearable devices combined with machine learning algorithms can analyze individual activities in real time, track treatment effects and assist medical staff to adjust treatment or management plans in time, so that patients can achieve better clinical results. Wearable sensor technology (55) by collecting physiological and behavioral data appears to hold promise to provide a proxy measure of cognitive function. Many MCI cases may not allow accurate and timely diagnosis, but technologies such as novel automated methods for early identification tracking and accurate MCI diagnosis and smart devices that combine machine learning algorithms promise to open new perspectives in bridging these aspects. In addition, machine learning also plays an extremely important role in the interdisciplinary data integration and personalized medicine in the MCI field. Machine learning can integrate data from different sources, such as neuroimaging, genetic, and behavioral data, to provide a more comprehensive view of the patient’s condition. Machine learning models that combine multidimensional data and cognitive test results perform better in predicting MCI progression than models using only a single data type. These advantages reflect the great potential of machine learning, which can not only help improve the quality of diagnosis and management of MCI, but also provide more meticulous and personalized care for patients (56).

### 4.2 Research hotspots

When exploring the risk assessment and efficient prediction of MCI, it is particularly noteworthy that recent attention has been raised in this field. Keyword analysis is helpful to understand the research topic, (Table 5) shows that the key keywords in this study are “Machine Learning,” “Mild Cognitive Impairment,” “Dementia,” “Classification,” “Diagnosis.” A further analysis from (Figures 6, 7) shows that the early identification, early diagnosis, early monitoring and early intervention of individual MCI with AI-based machine learning methods show the importance and urgent attention to this field.

Early accurate diagnosis technology deepening: Machine learning plays a key role in the early diagnosis of MCI. Using the fusion of multimodal data (such as neuroimaging, genomics, behavioral data, etc.), it can significantly improve the diagnostic accuracy. At present, the research hotspot in this field is to optimize the multimodal data fusion algorithm and mine the potential correlation between the data to achieve more accurate early identification. It can be seen from the text that the keywords “early diagnosis,” “computer-aided diagnosis” and “neuroimaging” appear more frequently, reflecting the researchers’ high attention to this direction. By integrating magnetic resonance imaging (MRI), positron emission tomography (PET), sleep electroencephalography (sleep EEG) and other imaging data, such as machine learning classification algorithms, it can effectively distinguish MCI patients from normal individuals, buying valuable time for early intervention.

Optimization of remote monitoring system: With the progress of technology, the remote monitoring system based on machine learning has become a research hotspot. With the help of physiological and behavioral data collected by wearable devices and smart home systems, machine learning algorithms can analyze changes in patients’ cognitive functions in real time, enabling remote and continuous monitoring. As mentioned in the article, that “continuous assessment and remote monitoring of cognitive function” is one of the current research directions, such as using the data collected by wearable sensors to predict the disease progression of MCI patients, help medical staff to adjust the treatment plan in time, improve the quality of life of patients, and reduce the burden of medical resources.

Joint model and dynamic prediction: In-depth evaluation of the same group at different time points to capture the changing trajectory of object cognitive function is one of the core themes today. In light of the practical challenges in conducting a full-cycle risk assessment for cognitive function objects, an alternative and highly promising area for exploration is the development of longitudinal warning models. These models, powered by advanced machine learning algorithms, integrate cross-sectional warning results from multiple time points, offering a novel approach beyond traditional cross-sectional alerts. Existing studies are commonly seen in combined models that combine longitudinal cognitive function assessment and survival analysis. While time-dependent covariates may vary over time and have an impact on MCI progression, the joint model equally gains the way to achieving dynamic prediction of MCI progression risk.

### 4.3 Feature trends

Multidisciplinary integration deepens: Machine learning research in the MCI field will rely more on the deep integration of multiple disciplines. In the future, biology, medicine and computer science will work closely together to build a more complete predictive model of cognitive impairment. By integrating biological markers and machine learning algorithms, it is expected to find more specific MCI predictors and improve the accuracy and reliability of prediction. Combined with the research results of neuroscience on the cognitive mechanism of the brain, the structure and algorithm of the machine learning model are optimized to make it more in line with the cognitive process of the brain and provide a more scientific basis for the diagnosis and treatment of MCI.

Innovation driven by AI technology: The continuous innovation of AI technology will bring new opportunities for the application of machine learning in MCI. In the future, more advanced deep learning algorithms, such as models based on transformer architecture, may be widely used in the study of MCI. These algorithms are able to better handle complex multimodal data, mine deep features in the data, and further improve the performance of disease prediction and diagnosis. Research progress in AI technology in interpreting model results will help to solve the problem of interpretability of machine learning models, enhance the trust of doctors and patients in model prediction results, and promote the widespread application of machine learning in clinical practice.

In conclusion, this study analyzes the literature on the topics of “machine learning” and “mild cognitive impairment” published over the past 10 years, mainly using bibliometric methods, and identifies countries, institutions, authors and journals that have made significant contributions to the field during this period. By analyzing the data in the research and presenting them in a visual way, we can better understand the role and status of different countries, institutions, authors and journals in scientific research cooperation, as well as the geographical distribution and connection intensity of scientific research cooperation from the macro, meso and micro perspectives based on the global scientific research cooperation network. This is of great significance to the formulation of scientific research cooperation strategies and the promotion of international scientific research exchanges. At present, the research in this field mainly focuses on feature extraction with the use of various machine learning algorithms to explore the prediction of MCI, automated monitoring of MCI, continuous evaluation and remote monitoring of cognitive function in MCI individuals, and the combination of interdisciplinary data integration with personalized medicine. Despite these advantages, potential challenges such as the interpretability of models and the need for high-quality training data are also questions worth pondering. Although the challenges are there, we believe that with our joint efforts, machine learning promises to create a bright future for people with MCI.

## 5 Limitations

Compared with traditional reviews, bibliometric-based analysis provides a broader perspective on the focus and developmental trends of machine learning in the MCI research field of MCI. But the study also has its limitations. To start with, this study only included literature from the WoSCC database, excluding data from other databases (e.g., PubMed and Scopus, etc.). Subsequently, only English literature was included, and papers in other languages failed to be included. Following the second point, due to the continuous updating of Wo SCC, the retrieval results of this study may not reflect the most recent literature. For the above reasons, there may be some degree of missing and biased data.
